# Functional divergence of Heat Shock Factors (Hsfs) during heat stress and recovery at the tissue and developmental scales in C4 grain amaranth (*Amaranthus hypochondriacus*)

**DOI:** 10.3389/fpls.2023.1151057

**Published:** 2023-04-11

**Authors:** Komal Goel, Pravesh Kundu, Vijay Gahlaut, Paras Sharma, Ayush Kumar, Shiwali Thakur, Vipasha Verma, Bhavya Bhargava, Rahul Chandora, Gaurav Zinta

**Affiliations:** ^1^ Biotechnology Division, CSIR-Institute of Himalayan Bioresource Technology, Palampur, Himachal Pradesh, India; ^2^ Academy of Scientific and Innovative Research (AcSIR), Ghaziabad, Uttar Pradesh, India; ^3^ Department of Biotechnology and University Center for Research and Development, Chandigarh University, Mohali, Punjab, India; ^4^ Agrotechnology Division, CSIR-Institute of Himalayan Bioresource Technology, Palampur, Himachal Pradesh, India; ^5^ ICAR-National Bureau of Plant Genetic Resources Regional Station, Shimla, Himachal Pradesh, India

**Keywords:** heat stress, Hsfs, underutilized crops, climate change, gene expression

## Abstract

Two major future challenges are an increase in global earth temperature and a growing world population, which threaten agricultural productivity and nutritional food security. Underutilized crops have the potential to become future climate crops due to their high climate-resilience and nutritional quality. In this context, C4 pseudocereals such as grain amaranths are very important as C4 crops are more heat tolerant than C3 crops. However, the thermal sensitivity of grain amaranths remains unexplored. Here, *Amaranthus hypochondriacus* was exposed to heat stress at the vegetative and reproductive stages to capture heat stress and recovery responses. Heat Shock Factors (Hsfs) form the central module to impart heat tolerance, thus we sought to identify and characterize Hsf genes. Chlorophyll content and chlorophyll fluorescence (Fv/Fm) reduced significantly during heat stress, while malondialdehyde (MDA) content increased, suggesting that heat exposure caused stress in the plants. The genome-wide analysis led to the identification of thirteen *AhHsfs*, which were classified into A, B and C classes. Gene expression profiling at the tissue and developmental scales resolution under heat stress revealed the transient upregulation of most of the Hsfs in the leaf and inflorescence tissues, which reverted back to control levels at the recovery time point. However, a few Hsfs somewhat sustained their upregulation during recovery phase. The study reported the identification, physical location, gene/motif structure, promoter analysis and phylogenetic relationships of Hsfs in *Amaranthus hypochondriacus*. Also, the genes identified may be crucial for future gene functional studies and develop thermotolerant cultivars.

## Introduction

A current trend in weather events and the increased food demands are the major concerns of today’s world. Among the various abiotic stresses that affect crop growth and development, heat stress is a serious threat to plant’s yield and economy (Wang et al., 2003). High temperature negatively impacts the morphology, physiology and metabolism of plants (Wahid et al., 2007). High-temperature susceptibility varies at the developmental level, various studies highlighted the reproductive phase as the most sensitive compared to the vegetative phase (Zinn et al., 2010). To cope with these environmental stresses, plants have developed various defense and signaling mechanisms. These include various transcriptional factors (TF), such as heat shock transcription factors (Hsfs) involved in resistance to heat and other abiotic stresses (Scharf et al., 2012). Hsfs form the central module of heat stress signaling network and mediate the expression of heat-responsive genes to activate the defense pathway. Heat Shock Proteins (HSPs) are one of the primary targets of Hsfs that get accumulated under heat stress, which act as molecular chaperones to maintain and restore protein homeostasis (Wahid et al., 2007).

Heat stress impact on plant is varied, and severe heat could leads to the collapse of cellular organization (Ahuja et al., 2010). Heat stress affects plant development by disrupting metabolic balance, protein, membranes and RNA stability, and by altering the enzymatic efficiency and cytoskeleton structure (Mcclung and Davis, 2010; Mittler and Blumwald, 2010; Lobell et al., 2011). Photosynthesis is considered one of the most heat-sensitive process in plants (Crafts-Brandner and Salvucci, 2002). High temperature greatly impact the photosynthetic capacity by altering the thylakoid structural organization and reducing the photosystem II (PSII) activity (Morales et al., 2003; Rodríguez et al., 2005). Heat markedly alters the photosynthetic pigment concentration as well (Marchand et al., 2005). Various metabolic pathways and enzymes are sensitive to heat stress and ROS (Reactive Oxygen Species) is accumulated which leads to oxidative stress (Asada, 2006). Heat stress-induced oxidative stress is responsible for membrane and protein degradation, enzyme deactivation and membrane lipid peroxidation (Savicka and Škute, 2010), resulting in malondialdehyde (MDA) accumulation (Hurkman et al., 2009).

Hsfs are structurally and functionally conserved in eukaryotic organisms (Lyck et al., 1997). The conserved domains like the DNA binding domain and oligomerization domain are the characteristic elements of Hsfs. At the N-terminal, the DNA binding domain (DBD) consists of antiparallel four-stranded β-sheets packed against a bundle of three α-helices. The hydrophobic core ensures the helix-turn-helix structure necessary for the recognition of heat shock promoter element motifs (Harrison et al., 1994). Adjacent to the DBD is another oligomerization domain (HR-A/B) comprised of hydrophobic heptad repeats, which form a helical coiled-coil structure responsible for trimerization of Hsfs and is separated from DBD by a flexible linker of variable lengths (Peteranderl et al., 1999). Based on the peculiarities of flexible linkers and the HR-A/B region, Hsfs families fall into three classes, namely A, B and C. Class A and C have an additional residue of 21 and 7 amino acids, respectively in their HR-A/B regions, while class B differs from class A and C by the absence of insertional residue (Lyck et al., 1997). Apart from the DBD and OD, there are other domains called NLS, and NES, which mediate the crosstalk of Hsfs between the nucleus and cytoplasm (Kotak et al., 2004). NLS (Nuclear localization signals) domain contains arginine and lysine, which serve as a signal for nuclear import. In contrast, the NES (Nuclear export signal) is hydrophobic and leucine-rich required for nuclear export. At the C-terminal, Class A Hsfs have another region named AHA motifs, which are hydrophobic and rich in aromatic residues and play a vital role in the activation of Hsfs. Unlike Class A, Class B and C have no activation role due to the lack of AHA motif (Lyck et al., 1997; Heerklotz et al., 2001; Kotak et al., 2004).

Grain Amaranth is a pseudocereal that belongs to Amaranthaceae and owns a great nutritional profile with high adaptability to adverse climates. It serves as a multipurpose crop whose grains and leaves are a source of nutritionally rich food, and because of its diverse color inflorescence, it can be used as an ornamental plant. *A. hypochondriacus* is a C4 annual herb native to Central and North America with a life cycle of 4-6 months and can grow in diverse geographic ranges (Joshi et al., 2018). They are self-pollinating diploids with a genome size of 500Mbp and 16, chromosome pairs (Lightfoot et al., 2017; Stetter et al., 2017). Amaranth possesses high adaptability because of the C4 photosynthesis pathway, which increases its CO_2_ utilization efficiency under high-temperature stress (Kigel, 2018). The seed of grain amaranth has a superior nutritional value than cereals (wheat, rice, maize) (Venskutonis and Kraujalis, 2013). Amaranth seeds have many essential amino acids, such as lysine and sulfur-containing amino acids (Juan et al., 2007; Mlakar et al., 2009).

Hsfs are master regulators of heat stress signaling pathways and form the central module to impart thermotolerance in plants. However, the Hsf gene family remains uncharacterized in Amaranth. We performed physiological analysis of *Amaranthus hypochondriacus* during heat stress exposure and recovery. Next, genome-wide identification, organization, structure, and promoter analysis of the Hsf gene family was performed, followed by temporal gene expression profiling in different tissues and developmental stages under heat stress and recovery. Our results provide valuable information for the functional characterization of the Hsf genes in *Amaranthus hypochondriacus*.

## Materials and methods

### Plant material and growth conditions

**Figure 1:**
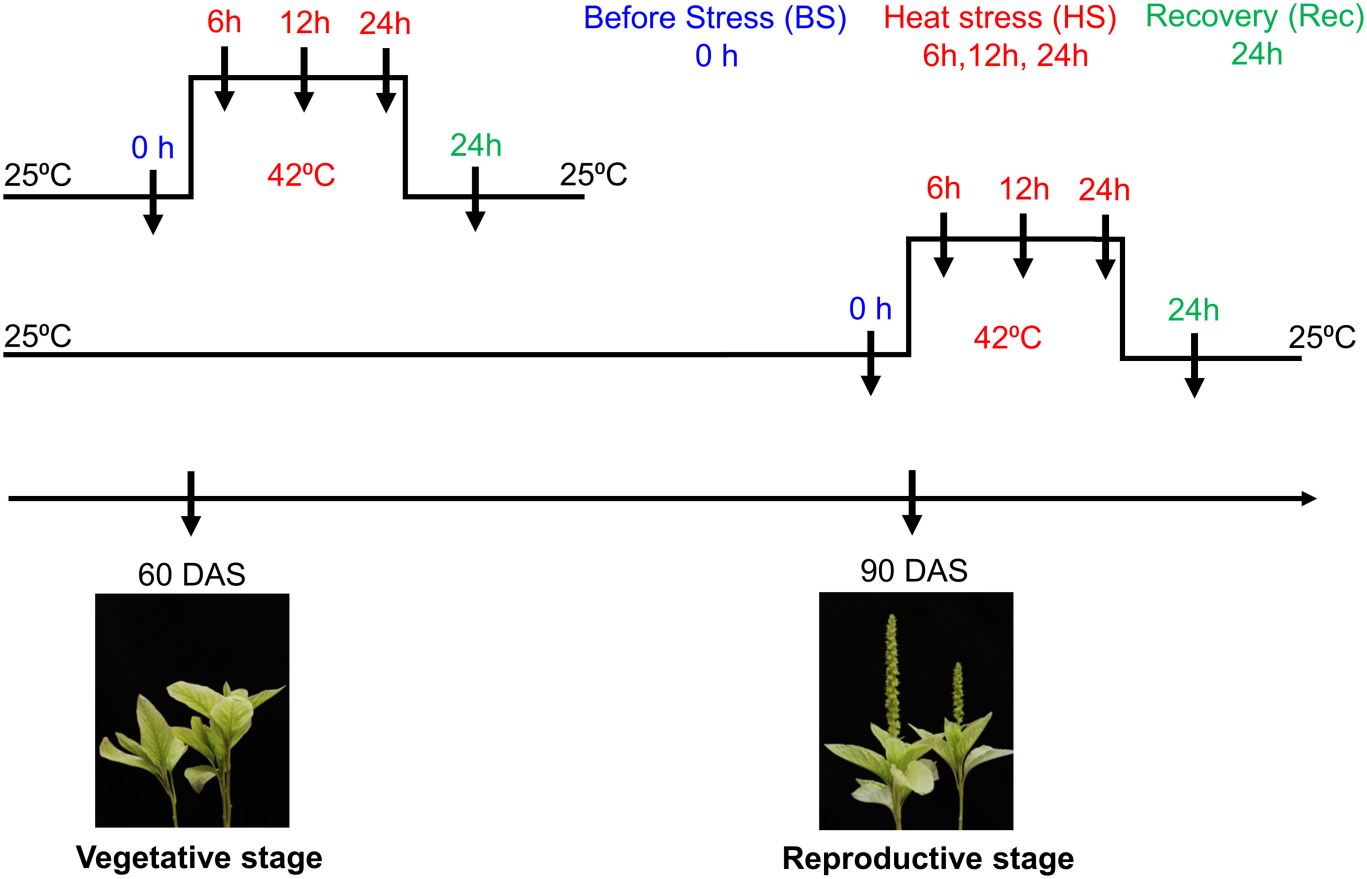
Schematic representation of *Amaranthus hypochondriacus* exposed to heat stress at vegetative (60 DAS) and reproductive (90 DAS) stages. Arrow represents different sampling time points i.e., before, during heat and recovery.

Seeds of *Amaranthus hypochondriacus* accession number “IC35589” were obtained from ICAR – National Bureau of Plant Genetic Resources – Regional Station, Shimla, Himachal Pradesh, India. Seeds were grown under controlled conditions in a walk-in Percival growth chamber (25 ± 1°C, 16-h light/8-h dark cycle). After four weeks, uniform-sized seedlings (two-leaf stage) were transplanted in pots containing soil mixture (coco peat and vermiculite 2:1). For heat stress, uniform-sized seedlings {true four leaf stage of 60 days after sowing (DAS)} at vegetative stage and at reproductive stage (90 DAS) were transferred to a growth chamber at 42 ± 1°C. Then, plants were shifted back to the control condition for the recovery of 24h. Then, the leaves and inflorescence tissues were collected for RNA extraction at heat stress and recovery time points (**Figure 1**). The harvested samples were frozen immediately in liquid nitrogen and stored at -80°C.

### Physiological and biochemical analysis

The chlorophyll fluorescence of PSII was monitored by using a portable JUNIOR-PAM fluorometer (PAM200, Waltz, Germany). First, the plant was dark adapted for 30 minutes, the initial fluorescence (Fo) was determined by the first low-intensity beam followed by maximal fluorescence (Fm). Thus, the data obtained was used to determine the Fv/Fm. To estimate the chlorophyll pigment, leaves were ground using a pestle and mortar, then extracted in a total volume of 1 ml of acetone 80% (v/v). The extract was centrifuged at 9000 g for 20 minutes. The supernatant was collected and absorbance was determined on a microplate reader at wavelengths of 470, 649 and 664 nm. The chlorophyll a, b and total carotenoid concentrations were calculated as described previously (Wellburn, 1994). For lipid peroxidation, MDA content was measured by homogenizing the 100 mg frozen sample in 1 ml ethanol 80% (v/v). The extract was centrifuged at 12000 g for 15 minutes. The 150 µl supernatant is added to 300 µl 0.5% TBA (Thiobarbituric acid) in 20% TCA. The reaction is heated for 1 hour at 90 °C and absorbance was taken at 532, 600 and 450 nm. The final quantity of MDA was calculated by using the formula: 6.45*(Abs_532_ – Abs_600_ – 0.56*Abs_450_).

### Identification and characterization of Hsfs in *A. hypochondriacus*

To identify the potential member of Hsf genes in Amaranth, the protein and nucleotide sequences of *Amaranthus hypochondriacus* were downloaded by using the conserved domain Hsf-type DBD domain (Pfam: PF00447) as a query in the BLAST search of *A. hypochondriacus* database of Phytozome and AmaranthGDB. To identify the putative Hsfs, the SMART7 (Schultz et al., 2000) software was used to recognize the conserved DBD domain and coiled-coil structure, which is the core of the (HR-A/B) domain. A distinct name was given to each of the Hsfs retrieved based on their scaffold location and distance from the centromere. The physical properties of the candidate proteins, such as amino acid length, molecular weight and protein isoelectric point (pI) were analyzed using the ProtParam tool of Expasy server https://www.expasy.org/(Artimo et al., 2012).

### Phylogenetic analysis

The Hsf proteins sequence of grain Amaranth along with *Arabidopsis thaliana*, *Chenopodium quinoa*, *Zea mays*, *Glycine max*, *Oryza sativa* were downloaded. Multiple sequence alignments were performed using Clustal W to compare protein sequences. A phylogenetic tree was constructed using the neighbor-joining (NJ) method with a bootstrap value of 1000 by MEGA 11 software (Tamura et al., 2021).

### Gene structure and domain analysis

The domain prediction was performed using different software tools. The SMART tool was used for the prediction of DBD and HR-A/B domains while the NLS domains were predicted using cNLS Mapper software (https://nls-mapper.iab.keio.ac.jp/cgi-bin/NLS_Mapper_form.cgi) and NES domains (http://prodata.swmed.edu/LocNES/LocNES.php) were identified using the LocNES tool. Likewise, the putative Hsfs conserved motifs were identified by submitting the protein sequences to MEME (Multiple Em for Motif Elicitation) software and further constructed using TBtool (Chen et al., 2018). The gene structure, including exons, introns and UTRs are displayed using the GSDS (Gene Structure Display Server) (Hu et al., 2015) software by submitting the FASTA file of CDS and genomic sequences.

### Cis-acting element analysis of *AhHsfs* and localization

The 1500-bp sequence upstream from the initiation codon of each gene was obtained from the *A. hypochondriacus* genome database. These sequences were used to identify cis-acting regulatory elements with the online software PlantCARE (Lescot et al., 2002). The subcellular localization of Hsf genes was determined by submitting the protein sequences in the web tool CELLO v.2.5 (Yu et al., 2014)

### RNA isolation and quantitative real-time PCR analyses

To confirm the expression of putative *AhHsf* genes, total RNA was isolated using Trizol reagent (Invitrogen, USA), followed by DNase I treatment by 1U/ul of DNase I (ThermoFisher Scientific Baltics, UAB, Lithuania) to remove any genomic DNA contamination. RNA concentration was determined by NanoDrop ND-1000 UV-Vis spectrophotometer (NanoDrop Technologies, Inc.), and the integrity of the RNA was assessed on a 1.2% (w/v) agarose gel. The first-strand cDNA was synthesized from 1 μg of total RNA using a verso cDNA synthesis kit (ThermoFisher Scientific Baltics, UAB, Lithuania). Quantitative RT-PCR was carried out using an Applied Biosystem manufactured step-plus real-time PCR system (Applied Biosystems, USA). Each reaction contains a 5.0 μL Dynamo Colorflash SYBR Green (ThermoFisher Scientific Baltics, UAB, Lithuania) 2.0 μL cDNA sample and 400 nM of gene-specific primer in a final volume of 10 μL. Each pair of primers were designed by using Primer 3 web software targeting an amplicon size of 100-200 bp. The primers used are listed in **Supplementary Table 1**. The thermal cycle used was as follows: 50°C for 2min, 95°C for 10 min, 40 cycles of 95°C for 15 s, and 60°C for 1 min. The specificity of the reactions was verified by melting curve analysis. The relative mRNA level for each gene was calculated as ^ΔΔ^CT values in comparison to unstressed plants (Applied Biosystems, USA). Amaranth MDH (Malate dehydrogenase) gene was used as an internal control for normalization. At least three replicates of each cDNA sample were performed for quantitative RT-PCR analysis.

### Statistical analysis

Analysis was performed using Excel 2019 (Microsoft, WA, USA), and graphs were plotted using PRISM 8 (GraphPad Software, CA, USA). Statistical differences were calculated by one-way analysis of variance (ANOVA) followed by Duncan’s test.

## Results

### Physiological and biochemical analysis

**Figure 2:**
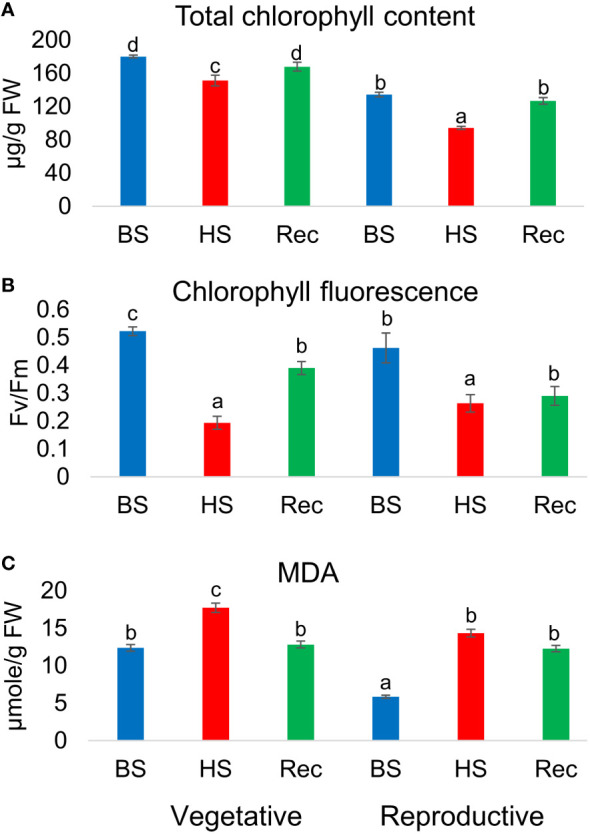
**(A)** Total chlorophyll content, **(B)** chlorophyll fluorescence (Fv/Fm), **(C)** malondialdehyde (MDA) content of *Amaranthus hypochonadriacus* performed at different sampling time points i.e. before (BS), heat (HS) and recovery (Rec) at vegetative (60 DAS) and reproductive (90 DAS) stages. Data were analysed using one-way ANOVA with *post hoc* Duncan’s test (letters indicate significant differences between the groups at p < 0.005). Error bars depict mean standard error.

Heat stress altered the total chlorophyll (Chl) content and leaf photochemical efficiency in the stressed plants. A reduction in total chlorophyll content (µg/g FW) was observed in heat stressed plants during development in relation to controls. However, during recovery the treated plants attain their initial level of total chlorophyll content in both the developmental stages. Similarly, chlorophyll fluoresecence or efficiency of photosystem ІІ in dark adapted leaves (Fv/Fm), were reduced in the stressed plants at the end of heat shock and were partially recovered. Estimation of lipid peroxidation revealed significantly high level of MDA in heat treated and recovery samples as compared to control **Figure 2**.

### Identification and physical locations of Hsf proteins in *A. hypochondriacus*

By using PF0047 (pfam ID) as a search query, we retrieved 19 Hsf as *AhHsf*s from *A. hypochondriacus* database. Subsequently, all Hsfs were surveyed for the presence of Hsf- type DBD domain, and 6 candidate genes were discarded due to the presence of an insignificant DBD domain. The physical and chemical properties of all 13 *AhHsf*s were analyzed (**Table 1**). The *AhHsf*s protein ranged from 202 aa (*AhHsf-2B*) to 505 aa (*AhHsf-7A*) in length. The predicted isoelectric point (pI) varied from 4.71 (*AhHsf*-*7A*) to 8.45 (*AhHsf-5B*) and molecular weight from 22.5 kDa (*AhHsf-2B*) to 55.8 kDa (*AhHsf*-*7A*). Further, the identified *AhHsf*s were named as per their scaffold location and the number of Hsfs on every scaffold varies. The largest number, comprised of three *AhHsf*s genes was found on scaffold 14 followed by scaffolds 7 and 9 with two Hsfs and the lowest number was found on scaffolds 2, 5, 12, 13, and 15 (one Hsf gene each).

**Table 1:** Protein information of amaranth Hsfs, including sequenced ID, protein sequence length, molecular weight (MW), isoelectric point (pI), and locations.

S.NO.	GENE	Gene ID	Amino acid residue	MW (Dalton)	pI	Location
1	*AhHsf-2B*	AH003731- HEAT STRESS TRANSCRIPTION FACTOR B-1	202	22578.32	5.78	Scaffold_2:24933467..24938004 forward
2	*AhHsf-4A*	AH007258- HEAT STRESS TRANSCRIPTION FACTOR A-1B-RELATED	493	55214.95	5.39	Scaffold_4:11317553..11324656 reverse
3	*AhHsf-5B*	AH008423- HEAT STRESS TRANSCRIPTION FACTOR B-4	271	31328.36	8.45	Scaffold_5:1165752..1167777 forward
4	*AhHsf-7A*	AH011529- HEAT STRESS TRANSCRIPTION FACTOR A-1A-RELATED	505	55850.97	4.71	Scaffold_7:10287911..10293172 forward
5	*AhHsf-7B*	AH012369- HEAT STRESS TRANSCRIPTION FACTOR B-2A	372	41683.74	5.33	Scaffold_7:23697020..23707371 reverse
6	*AhHsf-9C*	AH014540- HEAT STRESS TRANSCRIPTION FACTOR C-1	274	31660.6	6.27	Scaffold_9:14372146..14375412 reverse
7	*AhHsf-9A*	AH014784- E2F/DP FAMILY WINGED-HELIX DNA-BINDING DOMAIN-CONTAINING PROTEIN-RELATED	421	48508.16	5.85	Scaffold_9:17183579..17185249 forward
8	*AhHsf-12B*	AH018671- HEAT STRESS TRANSCRIPTION FACTOR B-2B	333	36214.09	5.23	Scaffold_12:7705291..7707355 forward
9	*AhHsf-13B*	AH019542- HEAT STRESS TRANSCRIPTION FACTOR B-2A	314	35207.59	7	Scaffold_13:6469400..6470966 reverse
10	*AhHsf-14C*	AH020851- HEAT STRESS TRANSCRIPTION FACTOR C-1	288	33055.07	6.36	Scaffold_14:9223742..9226960 forward
11	*AhHsf-14Aa*	AH021133- HEAT STRESS TRANSCRIPTION FACTOR A-6A-RELATED	337	39018.13	5.92	Scaffold_14:14574576..14577039 reverse
12	*AhHsf-14Ab*	AH021364- E2F/DP FAMILY WINGED-HELIX DNA-BINDING DOMAIN-CONTAINING PROTEIN-RELATED	355	40944.27	5.28	Scaffold_14:17074884..17076258 forward
13	*AhHsf-15A*	AH021848- HEAT STRESS TRANSCRIPTION FACTOR A-3	495	55213.75	4.98	Scaffold_15:1459510..1461794 reverse

### Phylogenetic analysis of *AhHsf*s

**Figure 3:**
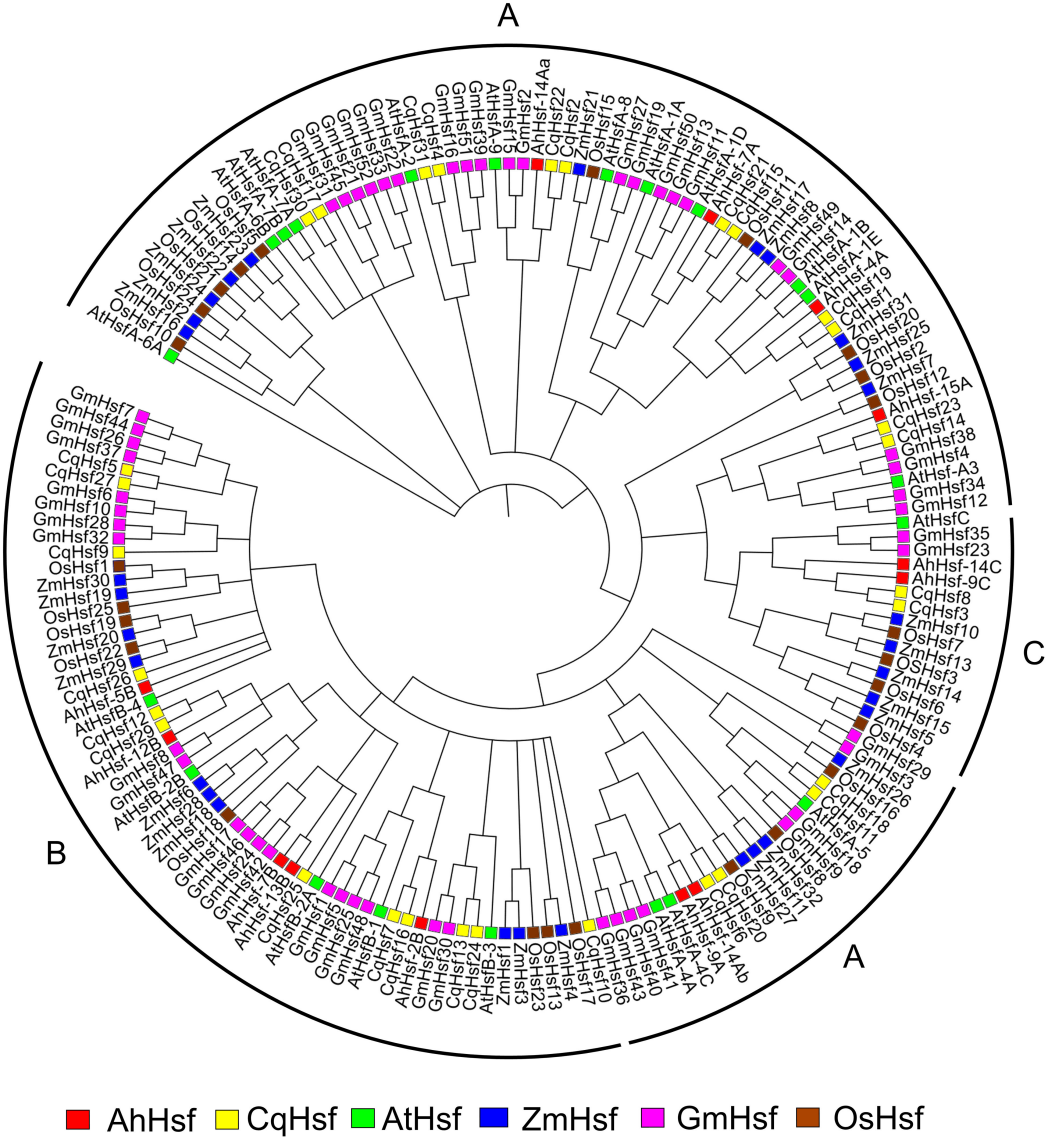
Phylogenetic tree depicting the evolutionary relationship of Hsf proteins of *A*. *hypochonadriacus* with *C. quinoa*, *A. thaliana*, Z. *mays*, *G. max*, *O. sativa* with 1000 bootstrap value.

To understand the phylogenetic relationship of Hsfs in Amaranth, all 13 *AhHsf*s protein sequences and the amino acid sequences of other species, *A. thaliana, Z. mays, C.quinoa, G.max, O.sativa* were used as a means to construct the phylogenetic tree. Hsfs genes from all the species were clustered into groups indicating their evolutionary events and biological functions were revealed by the homologous genes of the model plant **Figure 3**. All the Hsfs from all 6 species were divided into three classes (Class A, B and C) with well-supported bootstrap values, which include representative genes of *Arabidopsis*, maize, quinoa, soybean and rice. Out of 13 *AhHsf*s, 6 proteins belong to class A, making it the largest class, followed by classes B and C.

### Gene structure, conserved domains and motifs

**Figure 4:**
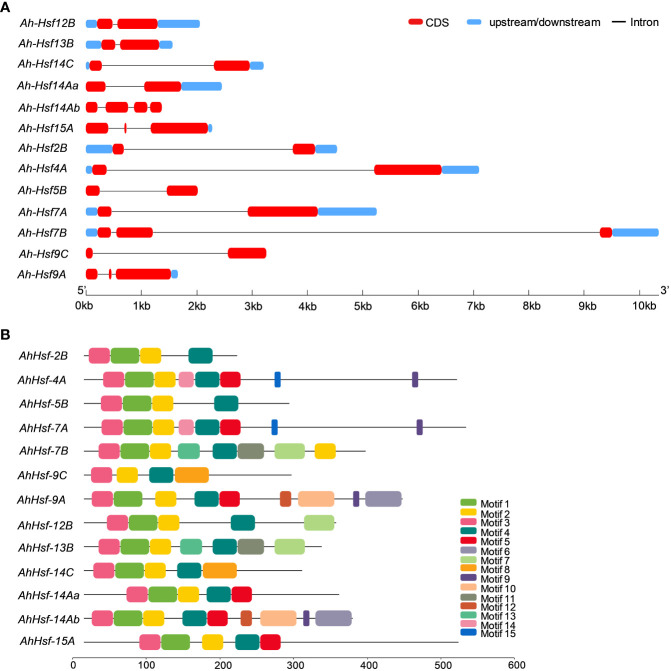
**(A)** Gene structure of *Hsfs* in *Amaranthus hypochondriacus*. The line represents the intron, and red and blue rectangular boxes represents CDS and upstream/downstream regions, respectively. **(B)** Distribution of conserved motifs in *Hsf* family member of *Amaranthus hypochondriacus*. The boxes represent the position and size of different motifs.

The structural diversity of the *AhHsf* family was analyzed in terms of the exon/intron arrangement of the coding sequences. The number of introns in *AhHsf*s ranged from one to three. The detailed gene structure of *AhHsf*s is shown in **Figure 4A**. MEME web server was employed to analyze motif distribution and verify the results of domain prediction.The motif distribution was consistent with the phylogenetic analysis as the members of the same class share some group-specific motifs apart from the conserved ones. Specifying the DBD domain & coiled-coil region, motifs 2, 3, and 4 were found in all the 13 Hsfs of Amaranth. NLS domain was completely absent from the class B Hsfs, whereas motif 5 and motif 8 were distinctly detected in class A and C Hsfs, respectively (**Figure 4B**; **Table 2**).

**Table 2:** Functional domains and motifs of amaranth Hsfs.

S.No.	Gene Name	DBD	HR-A/B (OD)	NLS	NES	AHA
1	*AhHsf-2B*	6-99	133-168	ND	163-177 (QCEELVGFLTAHLKV)	ND
2	*AhHsf-4A*	25-118	145-196	MP 225-238 (VTGVNKKRRLP) MP 228-237 (VNKKRRLPND)	406-420 (FTDSLSTVLNEALPM)	QTAGQRIQVMEQRQQQMMSFLAKAMHRP
3	*AhHsf-5B*	22-115	173-200	ND	208-222 (NYNSFKPVSYPFVGI)	ND
4	*AhHsf-7A*	23-116	145-196	BP 223-257 (RCISDANKKRRLKQDGVAESETSTLPDGQIVKYQP)	359-373 (ISGLVTPESISSVSL)	QTMVQRLQGMEQRQQQMMSFLAKAVQSP
5	*AhHsf-7B*	19-112	165-199	ND	243-257 (WGEDLQDLIDQMGFL)	ND
6	*AhHsf-9C*	9-68	85-118	MP 157-167 (VASPEKRRRLQ) MP 159-168 (SPEKRRRLQI)	223-237 (LLPLGFVPNLEHLQM)	ND
7	*AhHsf-9A*	10-119	142-195	MP 218-217 (NNRKRRVFRP) BP 218-246 (NNRKRRVFRPDHFYDETNNEDSSICTIRT)	91-105 (GKLILINGSLPMMIF)	QLLRDRLQQMERRQYSFASFCARRLQRP
8	*AhHsf-12B*	30-123	189-224	ND	212-226 (ELNHLRGLCNNILSL)	ND
9	*AhHsf-13B*	19-112	170-198	ND	188-202 (ELTSMKALCNNIFAL)	ND
10	*AhHsf-14C*	12-105	121-155	MP 194-204 (VSSPEKRRRLL) MP 196-205 (SPEKRRRLLM) BP 179-203 (DRDRAKHICGGAEVGVSSPEKRRRL)	41-55 (SFVVVEPLEFSQLIL)	ND
11	*AhHsf-14Aa*	56-149	157-193	MP 244-253 (GQVSKKRRLT) MP 246-254 (VSKKRRLTA) BP 119-148 (GFRKVHLDRWEFANSKFQRGKKHLLKTIKR)	170-184 (LRKEQEALQLEILDL)	ASMVDRIKSAEWKQREFIMLIAKAMKTT
12	*AhHsf-14Ab*	10-103	126-177	ND	207-221 (IDYELLEMMESSLHF)	QGLRDRLQHMERRQYSFASFFARALQKP
13	*AhHsf-15A*	73-181	198-237	ND	453-467 (PGEFSDVLDLGALQV)	ERVKDRLWASEQRQKQMVSFLAKVIQNP

MP, Monopartite NLS.

BP, Bipartite NLS.

ND, Not determined.

### Cis-acting element and expression patterns of Hsf genes in *A. hypochondriacus*

**Figure 5:**
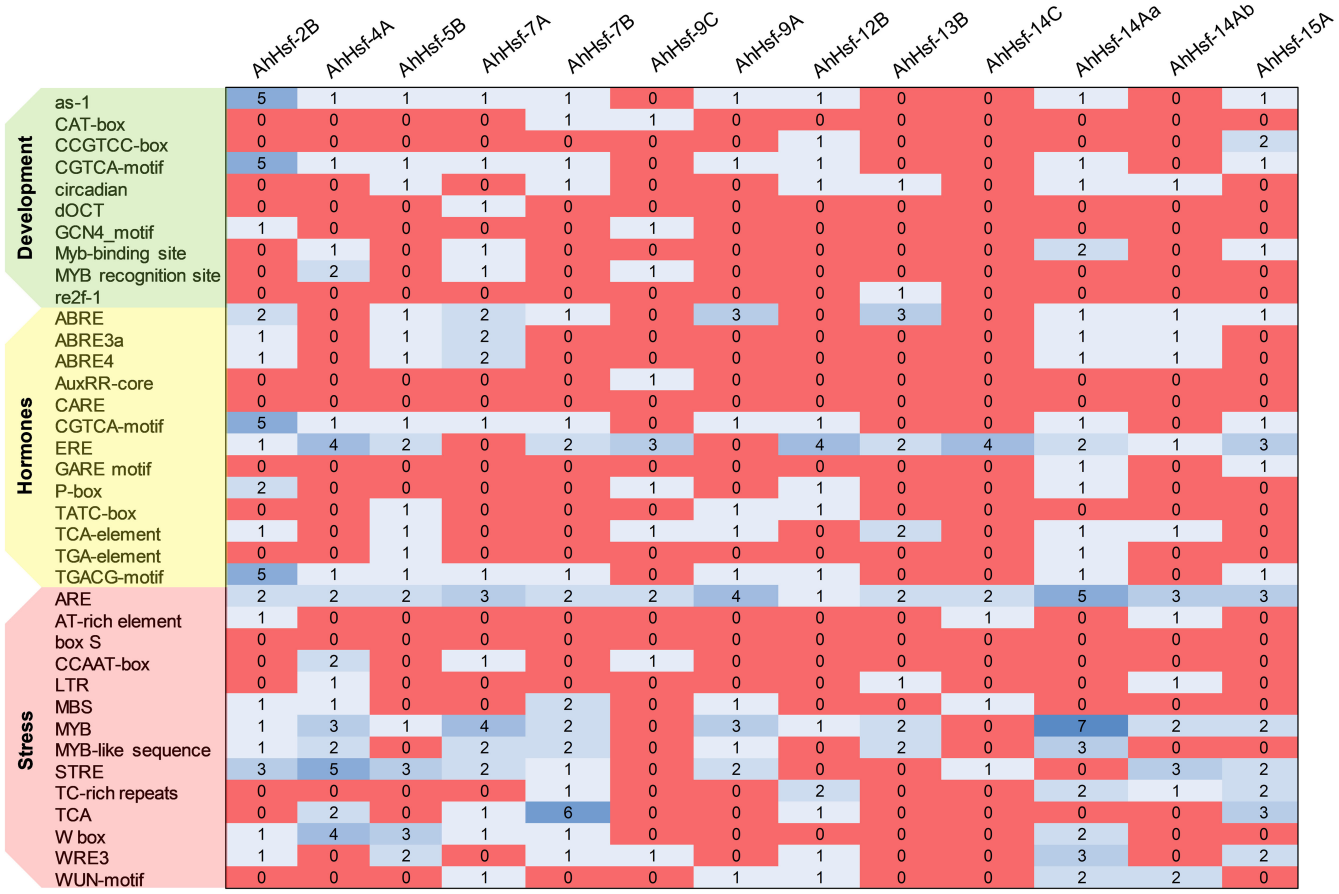
Cis-regulatory elements in the promoter region of *AhHsfs.* Based on the functional annotation, the cis-acting elements were classified into three major classes: stress, hormones, and development related cis-acting elements.

We analyzed the cis-acting element and observed that the promoter of each *AhHsf*s consists of various regulatory elements. As shown in the **Figure 5** the MYB element, ARE elements, STRE elements, TCA elements, ABRE (ABA-responsive element), and W-box were majorly found in all 13 *AhHsf*s. However, CAT-box, re2f-1, WRE-3, AT-rich regions, CARE, and ERE were also reported. These findings demonstrate that *AhHsf*s might be associated with various process like developmental, hormonal and stress responses.

**Figure 6:**
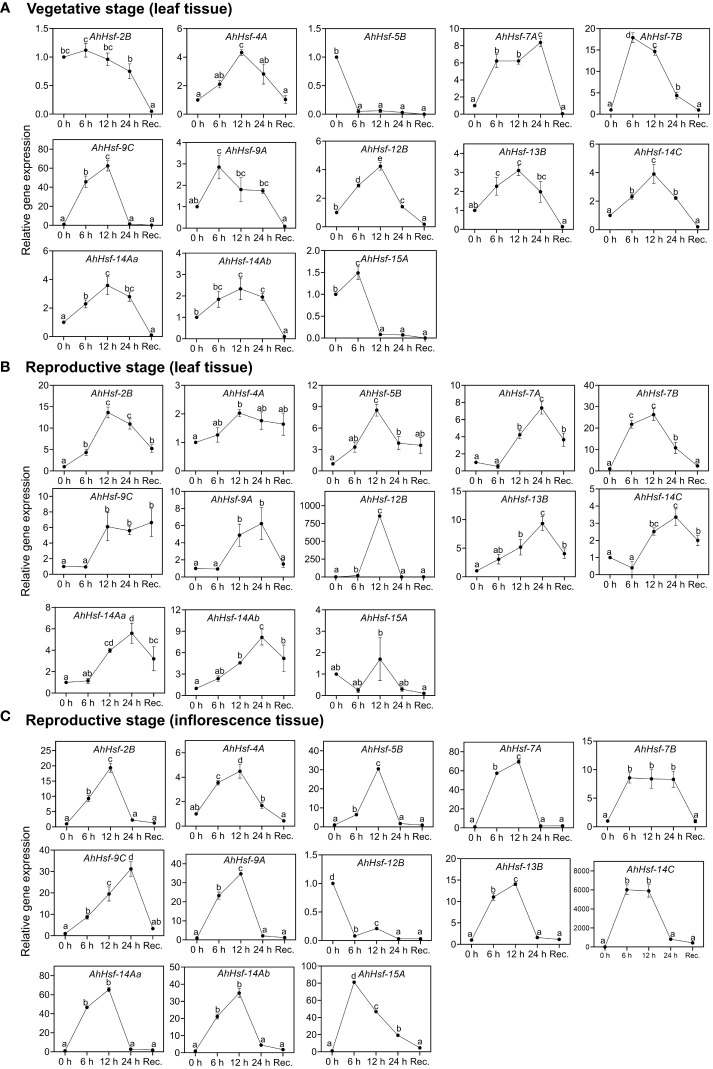
Relative gene expression level of *Hsfs* in the leaf and inflorescence tissues at the vegetative and reproductive stages of *A. hypochonadriacus* under heat stress and recovery time points. **(A)** leaf tissue at the vegetative stage, **(B)** leaf tissue at the reproductive stage, **(C)** inflorescence tissue at the reproductive stage. Three biological samples were measured with three technical replicates. The fold change in mRNA levels was calculated as the 2−ΔΔCt value relative to the mean values obtained in the control sample (set at a value of 1). Data were analysed using one-way ANOVA with *post hoc* Duncan’s tests (letters indicate significant differences between groups at p < 0.005). Error bars depict mean standard error.

To examine whether these predicted Hsfs were expressed in Amaranth during heat stress and recovery period, qRT-PCR was performed. The analysis revealed these genes expressed differentially in leaves of vegetative stage (**Figure 6A**), reproductive stage (**Figure 6B**) and inflorescence (**Figure 6C**) under heat stress and recovery phase. Most of the genes were strongly up-regulated on the onset of heat stress treatment and gradually decreased during the recovery. Three genes (*AhHsf*-2*B*, *Ah-Hsf-5B* and *AhHsf*-*15A*) were down-regulated during the heat stress in the vegetative phase while the opposite was observed in the reproductive stage. The same trend was observed in the *AhHsf-12B* in the inflorescence tissue of reproductive phase wherein this gene was down-regulated during and after heat stress (**Figure 6C**). Moreover, our results showed that the transcript level of all the Hsfs was highly elevated in the reproductive phase under heat stress. *AhHsf-7A, AhHsf-7B* and *AhHsf-9C* showed heat stress induction in all the developmental stages, stress time points and recovery.

## Discussion

Amaranth has gained enormous attention as an emerging pseudocereal because of its rich nutrient profile and presence of essential amino acid lysine (Písaříková et al., 2005). Apart from the nutritional quality, amaranth has wide geographical adaptability and can be grown in various environmental conditions. Hence, they are considered as potential future crops. Plants are exposed to variety of abiotic stresses such as high and low temperature, salinity, drought, and they employ various stress responsive mechanisms to combat the adversity (Mittler, 2006; Al-Whaibi, 2011). An interconnected network of mechanisms at physiological, biochemical and molecular levels is a prerequisite for the plant survival and adaptation during stress (Scharf et al., 2012). On the exposure of stress, transcription factors (TFs) converts the perceived signal to response mechanism by interacting with the cis-element of promoter region in the stress responsive genes followed by a cascade of gene interaction which increases the plant tolerance. This study provides insights on physiological, biochemical and functional divergence of Hsfs in grain amaranth (*Amaranthus hypochondriacus*).

Photosynthesis is the first process to get impaired on the exposure of high temperature (Camejo et al., 2005). However, decrease in photosynthesis is a result of structural and functional disruption of chloroplast, thylakoid membrane, reduction of chlorophyll accumulation, structural modification of PSII and excessive illumination (Dekov et al., 2000; Adir et al., 2003). High temperature causes structural damage of PSII reaction centre and phosphorylation of D1 protein which eventually degrades (Asada et al., 1998). Exposure of plants to high illumination generate reactive oxygen species (ROS) which inhibits the activity of photosystem II (PSII), an irreversible phenomenon called photoinhibition. Though plants are able to repair the PSII and overcome the photodamage, therefore the extent of photoinhibition depends on the balance between the damage and repair (Powles, 1984; Prasil et al., 1992). High temperature causes the accumulation of ROS which induces oxidative stress or injuries resulting in the enhancement of chlorophyll degradation, lipid peroxidation, decrease in antioxidant activity (Papadakis et al., 2004). In our study, we have observed the significant reduction in Fv/Fm ratio in the heat stressed plants confirming the occurrence of PSII damage and photoinhibition. The presence of photooxidative damage was also supported by the significantly higher MDA level and decreased chlorophyll content.

A comprehensive genome-wide identification of *AhHsf*s family was carried out and we identified 13 Hsfs in *Amaranthus hypochondriacus*, ranging from size of 1.5 kb 10 kb distributed on the scaffold of the amaranth genome. The difference observed in the physical and chemical properties of *AhHsf*s is due to the amino acid composition of non-conserved region. The widely accepted modular structure of Hsfs as reported by various studies defines the necessity of DBD and OD or coiled-coil structures (Scharf et al., 2012). Amaranth genome was analyzed for the identification of similar domain and all the five conserved domains were observed in majority of *AhHsf*s. Motif 2, 3 and 4 were found in all the 13 Hsfs of amaranth indicating toward the conserved domain DBD and HR-A/B. The DBD domain, the most conserved one is composed of 3 α-helices and 4 antiparallel β-sheets (Damberger et al., 1994). DBD possess a DNA-binding function and provides specific binding with heat shock promoter elements.The oligomerization domain (HR-A/B) which is a coiled-coil structure was also found in all the 13 Hsfs (Peteranderl et al., 1999). The balance of nuclear import and export is crucial for the intercellular distributions and interactions of Hsf genes. NES are the hydrophobic, leucine rich entites present at the C- terminal and is required for nuclear export along with AHA domain (Lyck et al., 1997). All 13 *AhHsf*s fall into three classes A,B and C as they belong to the same branch in accord with the evolutionary relationship of Arabidopsis (Nover et al., 2001). We observed that 6 Hsfs (*AhHsf-4A, AhHsf-7A, AhHsf-9A, AhHsf-14Aa, AhHsf-14Ab and AhHsf-15A*), 5 Hsfs (*AhHsf-2B, AhHsf-5B, AhHsf-7B, AhHsf-12B, AhHsf-13B*) and 2 Hsfs (*AhHsf-9C, AhHsf-14C*) belongs to class A, B and C, respectively. Interestingly, motif 5 was distinctly detected in all class A Hsfs representing AHA motif, however class B and C lacks AHA motifs as they do not have transcription activation ability.

Cis-elements in the promoter region regulate the gene expression of various metabolic pathways. The analysis of cis-regulatory elements in the promoter regions revealed that the amaranth Hsf genes contain MYB, MBS, ABRE, ARE, STRE, TC-rich elements, W-box, TCA, demonstrating they play significant role in regulation of stress response. STRE (stress responsive element) is the marker element of Hsfs protein located in the promoter region and have found in large number in most of the *AhHsf*s. Along with STRE, we found MYB binding sites which participates in drought, cold temperature and hormonal signalling (He et al., 2012). Other elements like ABRE, TCA-elements, DRE have role in regulating stress response (Liu et al., 2014). These finding suggests that *AhHsf*s could play role in plant stress responses. The expression pattern of *AhHsf*s in different tissue and developmental stages revealed their diverse function in amaranth suggesting towards their role in developmental as well as regulatory pathways. In this study, nearly all the *AhHsf*s were observed to be highly expressed in inflorescence tissue. As per the earlier studies done in *Arabidopsis* and tomato, class A Hsfs were defined as the master regulator for thermotolerance and have high potential to activate transcription of HSP genes (Mishra et al., 2002). Similarly, in our study, we found the up-regulation of *AhHsf-4A, AhHsf-7A, AhHsf-9A, AhHsf-14Aa, AhHsf-14Ab*, (Class A) during heat stress. *AhHsf-15A* was highly expressed in the inflorescence tissue, thus implying that function of *AhHsf-15A* might be conserved for the reproductive development regulation.

Class B Hsfs were reported to be involved in the development of reproductive organs and tissues. A study done in chickpea, demonstrated the upregulation of *CarHsfB2c* in the late flowering stages and *CarHsfB2a* in flower, pod and grain (Chidambaranathan et al., 2018). In amaranth, *AhHsf-2B, AhHsf-5B, AhHsf-7B, AhHsf-12B, AhHsf-13B* was up regulated in reproductive stage as compared to vegetative stage confirming its role in late developmental stages. As per the study done in *Vitis pseudoreticulata* where class C Hsfs level remained low in most of the tissues (Hu et al., 2016). Though in this work, we found the role of *AhHsf*-*9C* and *AhHsf*-*14C* in heat stress, suggesting class C Hsfs may have diverse function in different tissue.

**Figure 7:**
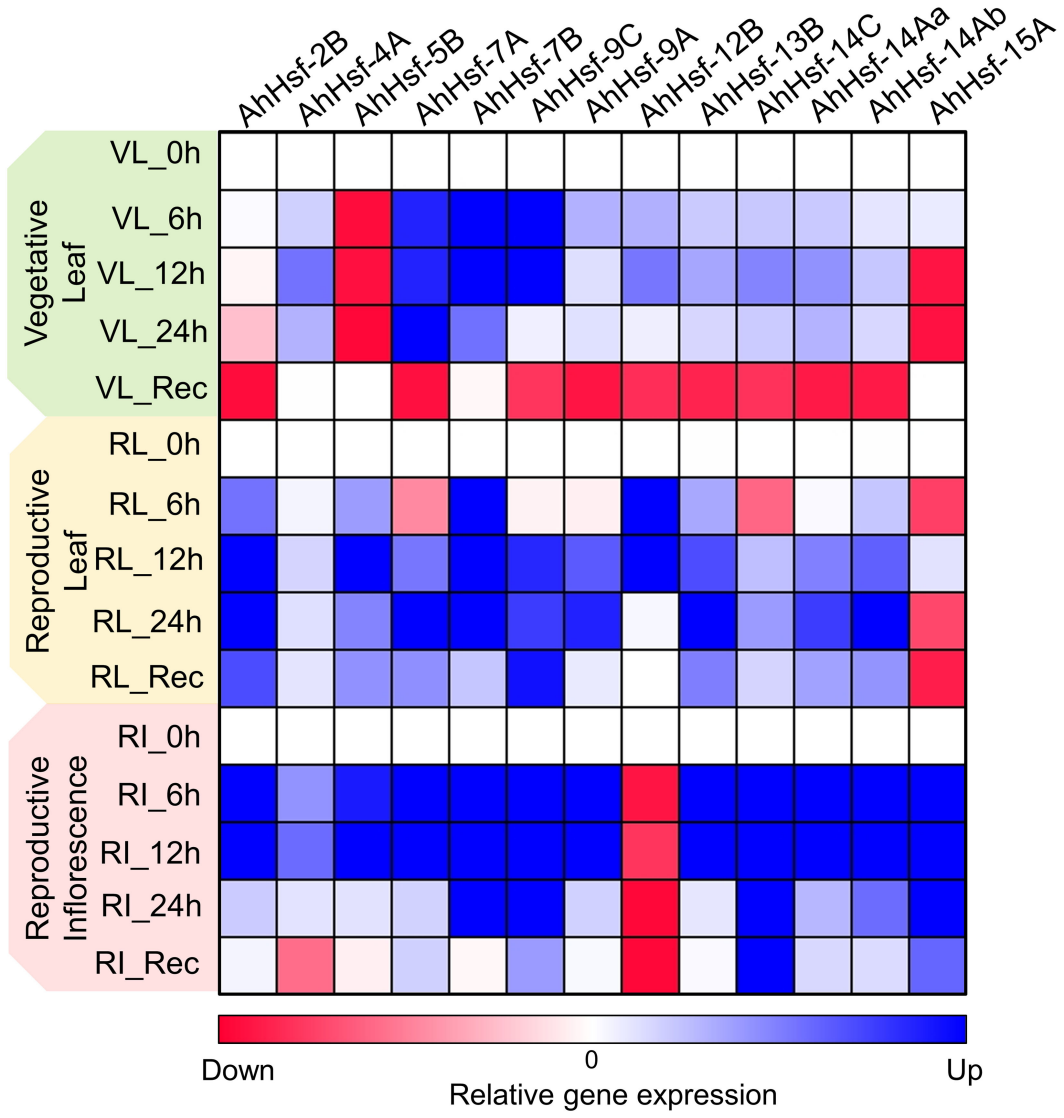
Heat map representation of temporal gene expression of amaranth *Hsfs* quantified at the vegetative and reproductive stages in leaf and inflorescence tissues under heat stress and recovery. The blue and red color indicates up regulation and down regulation of gene expression, respectively.

In our study we found that few Hsf genes exhibited distinct expression patterns in different tissues or organs, and were specific to stress duration and recovery (**Figure 7**). For example *AhHsf*-*2B* and *AhHsf*-*5B* specifically expressed in reproductive phase of plant development while *AhHsf*-*12B* and *AhHsf*-*15A* were specific to leaves and inflorescence tissue, respectively. Similarly, the recovery was prominent in reproductive phase as compared to the vegetative. Similar pattern of diverse gene expression in various tissue and at multiple developmental stages was observed in earlier studies done in maize and carnation (Lin et al., 2011; Li et al., 2019). Another study done in rice also displayed the tissue-specific expression of Hsfs (Liu et al., 2010). This tissue/organ-specific expression of Hsfs suggest the involvement of Hsfs in growth and development of plant.

Expression of Hsfs are said to be enhanced by high temperature (Swindell et al., 2007; Liu et al., 2009). The transcript level and time- course responses of each *AhHsf*s under heat stress exposure were distinct. The transcript level of *AhHsf*-*4A,9A,14Aa,14Ab* were up-regulated on the onset of heat stress, with the effect gradually diminishing during prolonged heat stress. While *AhHsf*-*7A,7B* and *9C* transcripts level increased and maintained at a relatively high level until 24 h. HSPs expressions are known to be controlled by Hsfs, while their accumulation could feed back to repress Hsf activity. This feedback inhibition could be one of the explanations behind the decline in expression of Hsfs during prolonged heat stress. Hsfs are responsible for transcription of heat stress responsive genes during heat stress and recovery. In this work, we found the gene expression of Hsfs are prominent in reproductive stage as compared to vegetative. Few Hsfs like *AhHsf*- *7A,9C, 14C, 14Aa* and *14Ab* showed up-regulated gene expression in recovery phase of leaf reproductive tissue. After stress, most regulatory responses tend to return to their prestress state while few develop some degree of memory to cope with the dynamic environment with repetitive stress (Crisp et al., 2016). The up-regulation of Hsfs in recovery phase at reproductive stage indicate their role in stress memory. Overall, this work reports the identification, physical location, gene/motif structure, promoter analysis and phylogenetic relationships of Hsfs in *Amaranthus hypochondriacus*. Such information is crucial for future gene functional studies in *A. hypochondriacus* and development of thermotolerant cultivars.

## Data availability statement

The original contributions presented in the study are included in the article/**Supplementary Material**. Further inquiries can be directed to the corresponding author.

## Author contributions

KG: Performed the experiments and *in silico* analysis, made figures and wrote the original draft; PK: Performed experiments and did *in silico* analysis; VG: *in silico* analysis and manuscript editing; PS: Performed experiments; AK: Performed experiments; ST: Performed experiments; VV: Performed experiments; BB: Contributed to resources*;* RC: Provided plant material; GZ: Conceived the idea, edited and finalized the manuscript. All authors contributed to the article and approved the submitted version.

## Supplementary material

Supplementary Table 1 Sequences of the primers used in this study.

## References

[B1] Adir N. Zer H. Shochat S. Ohad I. (2003). Photoinhibition–a historical perspective. Photosyn. Res. 76, 343–370. doi: 10.1023/A:1024969518145 16228592

[B2] Ahuja I. De Vos R. C. Bones A. M. Hall R. D. (2010). Plant molecular stress responses face climate change. Trends Plant Sci. 15, 664–674. doi: 10.1016/j.tplants.2010.08.002 20846898

[B3] Al-Whaibi M. H. (2011). Plant heat-shock proteins: a mini review. J. King Saud University-Science 23, 139–150. doi: 10.1016/j.jksus.2010.06.022

[B4] Artimo P. Jonnalagedda M. Arnold K. Baratin D. Csardi G. De Castro E.. (2012). ExPASy: SIB bioinformatics resource portal 40, W597–603. doi: 10.1093/nar/gks400 PMC3394269 22661580

[B5] Asada K. (2006). Production and scavenging of reactive oxygen species in chloroplasts and their functions. Plant Physiol. 141, 391–396. doi: 10.1104/pp.106.082040 16760493 PMC1475469

[B6] Asada K. Endo T. Mano J. Miyake C. (1998). “Molecular mechanism for relaxation of and protection from light stress,” in Stress responses of photosynthetic organisms. Eds. Saton K. Murata N. (Amsterdam: Elsevier).

[B7] Camejo D. Rodríguez P. Morales M. A. Dell’amico J. M. Torrecillas A. Alarcón J. J. (2005). High temperature effects on photosynthetic activity of two tomato cultivars with different heat susceptibility. J. Plant Physiol. 162, 281–289. doi: 10.1016/j.jplph.2004.07.014 15832680

[B8] Chen C. Chen H. He Y. Xia R. (2018). TBtools, a toolkit for biologists integrating various biological data handling tools with a user-friendly interface. BioRxiv 289660, 289660.

[B9] Chidambaranathan P. Jagannadham P. T. K. Satheesh V. Kohli D. Basavarajappa S. H. Chellapilla B.. (2018). Genome-wide analysis identifies chickpea (Cicer arietinum) heat stress transcription factors (Hsfs) responsive to heat stress at the pod development stage. J. Plant Res. 131, 525–542. doi: 10.1007/s10265-017-0948-y 28474118

[B10] Crafts-Brandner S. J. Salvucci M. E. (2002). Sensitivity of photosynthesis in a C4 plant, maize, to heat stress. Plant Physiol. 129, 1773–1780. doi: 10.1104/pp.002170 12177490 PMC166765

[B11] Crisp P. A. Ganguly D. Eichten S. R. Borevitz J. O. Pogson B. J. (2016). Reconsidering plant memory: Intersections between stress recovery, RNA turnover, and epigenetics. Sci. Adv. 2, e1501340. doi: 10.1126/sciadv.1501340 26989783 PMC4788475

[B12] Damberger F. F. Pelton J. G. Harrison C. J. Nelson H. C. Wemmer D. E. (1994). Solution structure of the DNA-binding domain of the heat shock transcription factor determined by multidimensional heteronuclear magnetic resonance spectroscopy. Protein Sci. 3, 1806–1821. doi: 10.1002/pro.5560031020 7849597 PMC2142621

[B13] Dekov I. Tsonev T. Yordanov I. (2000). Effects of water stress and high-temperature stress on the structure and activity of photosynthetic apparatus of zea mays and helianthus annuus. Photosynthetica 38, 361–366. doi: 10.1023/A:1010961218145

[B14] Harrison C. J. Bohm A. A. Nelson H. (1994). Crystal structure of the DNA binding domain of the heat shock transcription factor. Science 263, 224–227. doi: 10.1126/science.8284672 8284672

[B15] He Y. Li W. Lv J. Jia Y. Wang M. Xia G. (2012). Ectopic expression of a wheat MYB transcription factor gene, TaMYB73, improves salinity stress tolerance in arabidopsis thaliana. J. Exp. Bot. 63, 1511–1522. doi: 10.1093/jxb/err389 22140235

[B16] Heerklotz D. DoüRing P. Bonzelius F. Winkelhaus S. Nover L. (2001). The balance of nuclear import and export determines the intracellular distribution and function of tomato heat stress transcription factor HsfA2. Mol. Cell. Biol. 21, 1759–1768. doi: 10.1128/MCB.21.5.1759-1768.2001 11238913 PMC86729

[B18] Hu Y. Han Y.-T. Zhang K. Zhao F.-L. Li Y.-J. Zheng Y.. (2016). Identification and expression analysis of heat shock transcription factors in the wild Chinese grapevine (Vitis pseudoreticulata). Plant Physiol. Biochem. 99, 1–10. doi: 10.1016/j.plaphy.2015.11.020 26689772

[B17] Hu B. Jin J. Guo A.-Y. Zhang H. Luo J. Gao G. (2015). GSDS 2.0: an upgraded gene feature visualization server. Bioinformatics 31, 1296–1297. doi: 10.1093/bioinformatics/btu817 25504850 PMC4393523

[B19] Hurkman W. J. Vensel W. H. Tanaka C. K. Whitehand L. Altenbach S. B. (2009). Effect of high temperature on albumin and globulin accumulation in the endosperm proteome of the developing wheat grain. J. Cereal Sci. 49, 12–23. doi: 10.1016/j.jcs.2008.06.014

[B20] Joshi D. C. Sood S. Hosahatti R. Kant L. Pattanayak A. Kumar A.. (2018). From zero to hero: the past, present and future of grain amaranth breeding. Theor. Appl. Genet. 131, 1807–1823. doi: 10.1007/s00122-018-3138-y 29992369

[B21] Juan R. Pastor J. Alaiz M. Vioque J. (2007). Electrophoretic characterization of amaranthus l. seed proteins and its systematic implications. Bot. J. Linn. Soc. 155, 57–63. doi: 10.1111/j.1095-8339.2007.00665.x

[B22] Kigel J. (2018). “Development and ecophysiology of amaranths,” in Amaranth biology, chemistry, and technology (CRC Press), 39–73.

[B23] Kotak S. Port M. Ganguli A. Bicker F. Von Koskull-Döring P. (2004). Characterization of c-terminal domains of arabidopsis heat stress transcription factors (Hsfs) and identification of a new signature combination of plant class a hsfs with AHA and NES motifs essential for activator function and intracellular localization. Plant J. 39, 98–112. doi: 10.1111/j.1365-313X.2004.02111.x 15200645

[B24] Lescot M. Dé hais P. Thijs G. Marchal K. Moreau Y. Van de Peer Y.. (2002). PlantCARE, a database of plant cis-acting regulatory elements and a portal to tools for in silico analysis of promoter sequences. Nucleic Acids Res. 30, 325–327. doi: 10.1093/nar/30.1.325 11752327 PMC99092

[B25] Li W. Wan X.-L. Yu J.-Y. Wang K.-L. Zhang J. (2019). Genome-wide identification, classification, and expression analysis of the hsf gene family in carnation (Dianthus caryophyllus). Int. J. Mol. Sci. 20, 5233. doi: 10.3390/ijms20205233 31652538 PMC6829504

[B26] Lightfoot D. Jarvis D. E. Ramaraj T. Lee R. Jellen E. Maughan P. (2017). Single-molecule sequencing and Hi-c-based proximity-guided assembly of amaranth (Amaranthus hypochondriacus) chromosomes provide insights into genome evolution. BMC Biol. 15, 1–15. doi: 10.1186/s12915-017-0412-4 28854926 PMC5577786

[B27] Lin Y.-X. Jiang H.-Y. Chu Z.-X. Tang X.-L. Zhu S.-W. Cheng B.-J. (2011). Genome-wide identification, classification and analysis of heat shock transcription factor family in maize. BMC Genomics 12, 1–14. doi: 10.1186/1471-2164-12-76 PMC3039612 21272351

[B30] Liu J.-H. Peng T. Dai W. (2014). Critical cis-acting elements and interacting transcription factors: key players associated with abiotic stress responses in plants. Plant Mol. Biol. Rep. 32, 303–317. doi: 10.1007/s11105-013-0667-z

[B29] Liu J.-G. Qin Q.-L. Zhang Z. Peng R.-H. Xiong A.-S. Chen J.-M.. (2009). OsHSF7 gene in rice, oryza sativa l., encodes a transcription factor that functions as a high temperature receptive and responsive factor. BMB Rep. 42, 16–21. doi: 10.5483/BMBRep.2009.42.1.016 19192388

[B28] Liu A.-L. Zou J. Zhang X.-W. Zhou X.-Y. Wang W.-F. Xiong X.-Y.. (2010). Expression profiles of class a rice heat shock transcription factor genes under abiotic stresses. J. Plant Biol. 53, 142–149. doi: 10.1007/s12374-010-9099-6

[B31] Lobell D. B. Schlenker W. Costa-Roberts J. (2011). Climate trends and global crop production since 1980. Science 333, 616–620. doi: 10.1126/science.1204531 21551030

[B32] Lyck R. Harmening U. Höhfeld I. Treuter E. Scharf K.-D. Nover L. (1997). Intracellular distribution and identification of the nuclear localization signals of two plant heat-stress transcription factors. Planta 202, 117–125. doi: 10.1007/s004250050110 9177056

[B33] Marchand F. L. Mertens S. Kockelbergh F. Beyens L. Nijs I. (2005). Performance of high Arctic tundra plants improved during but deteriorated after exposure to a simulated extreme temperature event. Global Change Biol. 11, 2078–2089. doi: 10.1111/j.1365-2486.2005.01046.x 34991289

[B34] Mcclung C. R. Davis S. J. (2010). Ambient thermometers in plants: from physiological outputs towards mechanisms of thermal sensing. Curr. Biol. 20, R1086–R1092. doi: 10.1016/j.cub.2010.10.035 21172632

[B35] Mishra S. K. Tripp J. Winkelhaus S. Tschiersch B. Theres K. Nover L.. (2002). In the complex family of heat stress transcription factors, HsfA1 has a unique role as master regulator of thermotolerance in tomato. Genes Dev. 16, 1555–1567. doi: 10.1101/gad.228802 12080093 PMC186353

[B36] Mittler R. (2006). Abiotic stress, the field environment and stress combination. Trends Plant Sci. 11, 15–19. doi: 10.1016/j.tplants.2005.11.002 16359910

[B37] Mittler R. Blumwald E. (2010). Genetic engineering for modern agriculture: challenges and perspectives. Annu. Rev. Plant Biol. 61, 443–462. doi: 10.1146/annurev-arplant-042809-112116 20192746

[B38] Mlakar S. G. Turinek M. Jakop M. Bavec M. Bavec F. (2009). Nutrition value and use of grain amaranth: potential future application in bread making. Agricultura 6, 43–53.

[B39] Morales D. Rodríguez P. Dell'amico J. Nicolas E. Torrecillas A. Sánchez-Blanco M. J. (2003). High-temperature preconditioning and thermal shock imposition affects water relations, gas exchange and root hydraulic conductivity in tomato. Biol. Plant. 47, 203–208. doi: 10.1023/B:BIOP.0000022252.70836.fc

[B40] Nover L. Bharti K. Döring P. Mishra S. K. Ganguli A. Scharf K.-D. (2001). Arabidopsis and the heat stress transcription factor world: how many heat stress transcription factors do we need? Cell Stress chaperones 6, 177. doi: 10.1379/1466-1268(2001)006<0177:AATHST>2.0.CO;2 11599559 PMC434399

[B41] Papadakis I. Dimassi K. Bosabalidis A. Therios I. Patakas A. Giannakoula A. (2004). Effects of b excess on some physiological and anatomical parameters of ‘Navelina’orange plants grafted on two rootstocks. Environ. Exp. Bot. 51, 247–257. doi: 10.1016/j.envexpbot.2003.11.004

[B42] Peteranderl R. Rabenstein M. Shin Y.-K. Liu C. W. Wemmer D. E. King D. S.. (1999). Biochemical and biophysical characterization of the trimerization domain from the heat shock transcription factor. Biochemistry 38, 3559–3569. doi: 10.1021/bi981774j 10090742

[B43] Písaříková B. Kráčmar S. Herzig I. (2005). Amino acid contents and biological value of protein in various amaranth species. Czech J. Anim. Sci. 50, 169–174. doi: 10.17221/4011-CJAS

[B44] Powles S. B. (1984). Photoinhibition of photosynthesis induced by visible light. Annu. Rev. Plant Physiol. 35, 15–44. doi: 10.1146/annurev.pp.35.060184.000311

[B45] Prasil O. Adir N. Ohad I. (1992). Dynamics of photosystem II: mechanism of photoinhibition and recovery process. Topics photosyn. 11, 295–348.

[B46] Rodríguez M. Canales E. Borrás-Hidalgo O. (2005). Molecular aspects of abiotic stress in plants. Biotecnol. Aplicada 22, 1–10.

[B47] Savicka M. Škute N. (2010). Effects of high temperature on malondialdehyde content, superoxide production and growth changes in wheat seedlings (Triticum aestivum l.). Ekologija 56, 26–33. doi: 10.2478/v10055-010-0004-x

[B48] Scharf K.-D. Berberich T. Ebersberger I. Nover L. (2012). The plant heat stress transcription factor (Hsf) family: structure, function and evolution. Biochim. Biophys. Acta (BBA)-Gene Regul. Mech. 1819, 104–119. doi: 10.1016/j.bbagrm.2011.10.002 22033015

[B49] Schultz J. Copley R. R. Doerks T. Ponting C. P. Bork P. (2000). SMART: a web-based tool for the study of genetically mobile domains. Nucleic Acids Res. 28, 231–234. doi: 10.1093/nar/28.1.231 10592234 PMC102444

[B50] Stetter M. G. Müller T. Schmid K. J. (2017). Genomic and phenotypic evidence for an incomplete domestication of south American grain amaranth (Amaranthus caudatus). Mol. Ecol. 26, 871–886. doi: 10.1111/mec.13974 28019043

[B51] Swindell W. R. Huebner M. Weber A. P. (2007). Transcriptional profiling of arabidopsis heat shock proteins and transcription factors reveals extensive overlap between heat and non-heat stress response pathways. BMC Genomics 8, 1–15. doi: 10.1186/1471-2164-8-125 17519032 PMC1887538

[B52] Tamura K. Stecher G. Kumar S. (2021). MEGA11: molecular evolutionary genetics analysis version 11. Mol. Biol. Evol. 38, 3022–3027. doi: 10.1093/molbev/msab120 33892491 PMC8233496

[B53] Venskutonis P. R. Kraujalis P. (2013). Nutritional components of amaranth seeds and vegetables: a review on composition, properties, and uses. Compr. Rev. Food Sci. Food Saf. 12, 381–412. doi: 10.1111/1541-4337.12021 33412681

[B54] Wahid A. Gelani S. Ashraf M. Foolad M. R. (2007). Heat tolerance in plants: an overview. Environ. Exp. Bot. 61, 199–223. doi: 10.1016/j.envexpbot.2007.05.011

[B55] Wang W. Vinocur B. Altman A. (2003). Plant responses to drought, salinity and extreme temperatures: towards genetic engineering for stress tolerance. Planta 218, 1–14. doi: 10.1007/s00425-003-1105-5 14513379

[B56] Wellburn A. R. (1994). The spectral determination of chlorophylls a and b, as well as total carotenoids, using various solvents with spectrophotometers of different resolution. J. Plant Physiol. 144, 307–313. doi: 10.1016/S0176-1617(11)81192-2

[B57] Yu C.-S. Cheng C.-W. Su W.-C. Chang K.-C. Huang S.-W. Hwang J.-K.. (2014). CELLO2GO: a web server for protein subCELlular LOcalization prediction with functional gene ontology annotation. PloS One 9, e99368. doi: 10.1371/journal.pone.0099368 24911789 PMC4049835

[B58] Zinn K. E. Tunc-Ozdemir M. Harper J. F. (2010). Temperature stress and plant sexual reproduction: uncovering the weakest links. J. Exp. Bot. 61, 1959–1968. doi: 10.1093/jxb/erq053 20351019 PMC2917059

