# Supplementary material for: Functional divergence of Heat Shock Factors (Hsfs) during heat stress and recovery at the tissue and developmental scales in C4 grain amaranth (Amaranthus hypochondriacus)
Source: Front Plant Sci. 2023 Apr 11;14:1151057. doi: 10.3389/fpls.2023.1151057 (PMC10141669; doi:10.3389/fpls.2023.1151057)
Supplement: Supplementary Table 1 — Sequences of the primers used in this study. [file Table_1.docx]

| **S.no** | **Genes** | **Forward Primer** | **Reverse Primer** |
| --- | --- | --- | --- |
| 1 | *AhHsf-2B* | 5’ACATCCACATCATCACCGGG3’ | 5’TTATTTGATCCGGGCCCACC3’ |
| 2 | *AhHsf-4A* | 5’GAGCTGAGGGAGGAACATGG3’ | 5’CTGGATCTGGCGGAGGATTG3’ |
| 3 | *AhHsf-5B* | 5’TCAGTTCCAGCCCCATTTCT3’ | 5’ATGTGTTGAGCTGGCGAACA3’ |
| 4 | *AhHsf-7A* | 5’ATGAGGGGTTCTTGAGGGGT3’ | 5’TCCACCTCTTCCTCTAGCCC3’ |
| 5 | *AhHsf-7B* | 5’TGAGGGTAGCGGAAGTGAGA3’ | 5’ACATCACAGGGCTCGCATTT3’ |
| 6 | *AhHsf-9C* | 5’TGGTTCTTGCGAGGCCAAAA3’ | 5’ACTCGAGGAAGATGTGGATGA3’ |
| 7 | *AhHsf-9A* | 5’GTGCAGAGAAATCCGACCCA3’ | 5’GAGTCGAGAAGCAGGATCCG3’ |
| 8 | *AhHsf-12B* | 5’AGTTACGCCGGCAACAGTTA3’ | 5’AGAATCTCCGCCGTTGTTGT3’ |
| 9 | *AhHsf-13B* | 5’CAGGCGCTGGATTTGTTGAC3’ | 5’TGCACCTTCACCACACCTTT3’ |
| 10 | *AhHsf-14C* | 5’CGACCCCACCATCAACTTCA3’ | 5’ACACGTGGCAAGATCTCAGG3’ |
| 11 | *AhHsf-14Aa* | 5’GGCACTCCAACTCGAAATCC3’ | 5’ATCAGCTGTTGGAACGAGGG3’ |
| 12 | *AhHsf-14Ab* | 5’CGGACCCTGGACCAAATGAT3’ | 5’AGCTGCCCAATGTGTTCTGG3’ |
| 13 | *AhHsf-15A* | 5’CTCGAAGGGTAAACAGGCGT3’ | 5’AATGCAGGGCTTGAACTCGA3’ |
